# Silicon Seed Priming Mitigates Drought-Induced Effects on Growth, Water Status, and Photosystem Activity in Maize

**DOI:** 10.3390/plants15142174

**Published:** 2026-07-15

**Authors:** Yosra Ibrahim, Hasna Ellouzi, Farah Bounaouara, Rabaa Hidri, Mokded Rabhi, Ahmed Debez, Chedly Abdelly, Walid Zorrig

**Affiliations:** 1 Laboratory of Extremophile Plants, Centre of Biotechnology of Borj-Cedria, P.O. Box 901, Hammam-Lif 2050, Tunisia; yosra.ibrahim@cbbc.rnrt.tn (Y.I.); hasna.ellouzi@cbbc.rnrt.tn (H.E.); farah.bounaouara@cbbc.rnrt.tn (F.B.); rabaa.hidri@cbbc.rnrt.tn (R.H.); m.rabhi@qu.edu.sa (M.R.); ahmed.debez@cbbc.rnrt.tn (A.D.); chedly.abdelly@cbbc.rnrt.tn (C.A.); 2 Department of Plant Production, College of Agriculture and Food, Qassim University, Buraydah 52571, Saudi Arabia

**Keywords:** drought, silicon, seed priming, *Zea mays*, photosynthetic activity, photosystem II, photosystem I

## Abstract

Water deficit is a major abiotic constraint limiting maize growth and productivity worldwide. Although silicon (Si) is not classified as an essential element, its beneficial effects on numerous crop species are well documented. Silicon has been shown to promote plant growth and enhance tolerance to abiotic stresses, particularly drought stress. Seed priming, a pre-sowing technique known to stimulate early germination processes, has emerged as a promising approach to enhance seedling establishment and stress tolerance in crops. In the present study, silicon-based seed priming was investigated as a strategy to alleviate the adverse effects of water deficit in maize (*Zea mays*) using two sodium silicate priming-solution concentrations (10 and 20 mM). Maize plants were subjected to six experimental treatments based on seed priming: three under well-watered conditions (no silicon seed priming and seed priming with 10 and 20 mM sodium silicate solutions) and three corresponding treatments combined with irrigation withdrawal for 15 days to induce drought stress. Morphological traits, biomass accumulation, photosynthetic pigment content, plant water status, and PSI- and PSII-related photochemical parameters were evaluated. Drought stress markedly reduced plant growth, biomass production, relative water content, chlorophyll pigment levels, and photosystem photochemical performance, reflecting a strong negative impact of water deficit on most measured parameters. In particular, root and shoot fresh weights decreased by 75% and 71%, respectively, compared with those of well-watered unprimed control plants, indicating a substantial reduction in biomass accumulation under drought conditions. Furthermore, drought conditions impaired photochemical performance and increased non-regulated energy dissipation, indicative of impaired photosynthetic performance. Silicon seed priming mitigated several drought-induced effects in a trait-dependent manner. Under water-deficit conditions, 20 mM Si produced the strongest improvement in root and shoot fresh weights, whereas 10 mM Si showed stronger responses for selected shoot-growth and PSI-related parameters. Both Si treatments improved leaf water status and photosynthetic stability to varying extents. Collectively, these results indicate that sodium silicate seed priming partially improves drought-related responses in maize seedlings under the conditions of this study by sustaining growth performance, preserving plant water status, and maintaining photosynthetic stability under water-deficit conditions.

## 1. Introduction

Drought stress represents one of the most critical environmental factors limiting crop productivity, particularly in arid and semi-arid regions. Rising temperatures accelerate soil desiccation, leading to water stress that severely impairs plant growth and productivity [1,2,3]. Drought exerts a profound effect on the global food supply, particularly on staple crops such as rice, wheat, and maize, which are essential for human nutrition and food security [4]. Water deficit poses a serious threat to agricultural production by inducing major physiological alterations in plants, ultimately resulting in growth reduction or complete growth arrest [5]. Drought can substantially reduce the productivity of staple crops such as rice, wheat, and maize, with the magnitude of yield loss depending on crop species, cultivars, developmental stage, drought timing, and stress intensity [4,5,6].

In response to rising temperatures and increasing drought intensity, scientific research has increasingly focused on regulatory mechanisms, breeding strategies, and beneficial elements, such as silicon, to improve plant tolerance to adverse conditions [7].

Although silicon is not considered an essential element, numerous studies have highlighted its beneficial role in enhancing stress tolerance and regulating the physiological and biochemical responses of plants subjected to water-deficit conditions. Silicon is the second-most abundant element in the Earth’s crust and is primarily found in soils and clays in the form of silica and silicates [8]. Amorphous silica contributes significantly to silicon dissolution in the soil solution due to its higher solubility relative to the crystalline form [9]. For plants, the form available in soil solutions is monosilicic acid (H_4_SiO_4_) [10]. Studies have demonstrated that silicon application can reduce plant sensitivity to drought, thereby increasing crop yields under drought and salinity conditions [11]. Silicon helps stabilize the photosynthetic apparatus and supports the functional activity of photosystems I and II under stress conditions [12], thereby contributing to plant performance under adverse environmental conditions [13,14,15]. In soybean, silicon application was reported to alleviate drought-induced damage by improving growth, physiological, biochemical, and root-related traits [16].

Seed priming has emerged as a useful strategy to improve early seedling establishment and stress responsiveness, partly through priming-induced physiological and metabolic adjustments during seed conditioning. Plants derived from primed seeds exhibit improved physiological and metabolic performance, resulting in enhanced vigor and a greater capacity to adapt to adverse environmental conditions [17]. According to Shahzad et al. [18], silicon-based seed priming under water stress conditions improved gas exchange, water relations, and plant growth in canola. The effectiveness of this approach has also been reported across several crop species, including maize [19], wheat [20], soybean [21], and faba bean [22]. Furthermore, seed priming promotes the regulation of primary and secondary metabolism, strengthens antioxidant defense systems, and activates osmoprotective and osmoregulatory mechanisms that collectively contribute to improved stress tolerance.

Silicon- or silicic-acid-based priming has been reported to improve physiological responses associated with drought tolerance in cowpea [23]. Silicon supplementation reduced the negative effects of drought stress on maize photosynthesis and biomass accumulation by enhancing stomatal conductance and electron transfer efficiency between PSII and PSI. By increasing the performance index for energy conservation from photons absorbed by PSII, silicon supply may modulate intersystem electron transport and reduce excessive energy dissipation, thereby protecting PSII against photo-oxidative damage under drought stress and resulting in significant improvements in maize photosynthetic recovery and grain yield [24]. However, despite these advances, the effects of silicon seed priming on the coordinated functioning of PSII and PSI in maize seedlings under water-deficit conditions remain insufficiently characterized. In particular, few studies have simultaneously assessed growth, water status, pigment preservation, and PSI/PSII photochemical responses to different sodium silicate priming-solution concentrations. This gap limits our understanding of whether silicon seed priming acts uniformly or induces trait- and photosystem-specific responses in maize under drought.

To address this knowledge gap, the present study was conducted to test two primary hypotheses. First, it was hypothesized that silicon seed priming may promote plant growth and optimize plant water status, thereby mitigating the deleterious effects of drought stress. Second, it was postulated that silicon seed priming could enhance the functional activity of both photosystems (PSI and PSII), thereby improving photosystem photochemical performance under water-deficit conditions.

Accordingly, the objective of the present study was to investigate the potential of silicon-based seed priming, applied as sodium silicate priming solutions at 10 and 20 mM, to alleviate drought-induced damage in maize (*Zea mays* L.). Although previous studies have reported beneficial effects of silicon seed priming in maize under drought stress, the present work provides an integrated evaluation of growth performance, fresh biomass, photosynthetic pigment composition, leaf relative water content, and, importantly, a detailed simultaneous assessment of PSII and PSI photochemical responses using Dual-PAM measurements. This photosystem-focused approach represents the main originality of the study and allows a more specific evaluation of trait- and photosystem-dependent responses to silicon seed priming under water-deficit conditions.

## 2. Results

### 2.1. Morphological Aspects

**Figure 1:**
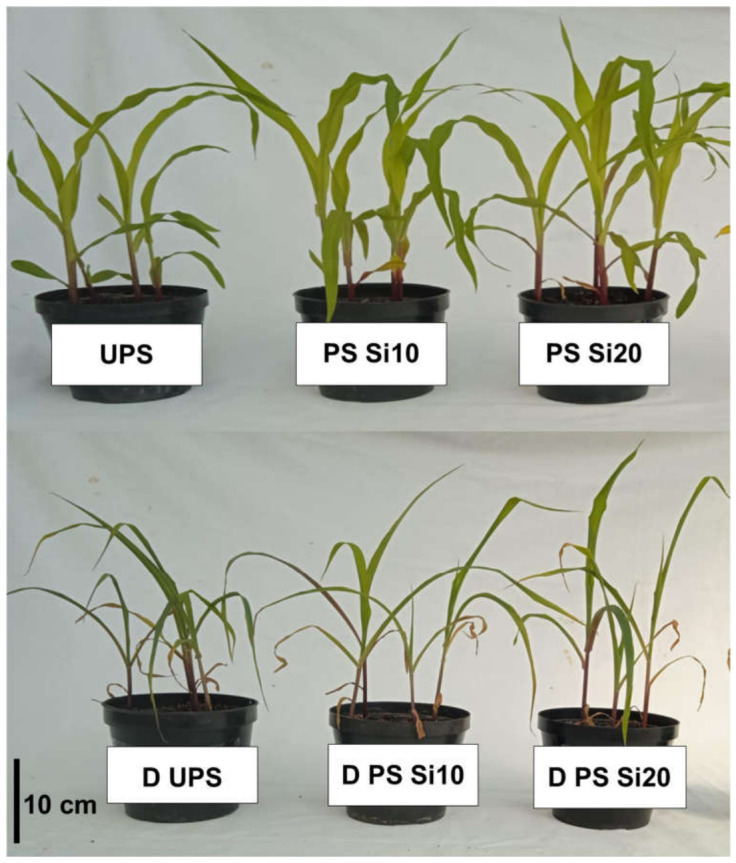
Phenotypic response of maize (*Zea mays* L.) plants subjected to water deficit with or without silicon seed priming. Plants were exposed to six treatments: three well-watered treatments comprising unprimed seeds and seeds primed with 10 or 20 mM sodium silicate, and three corresponding water-deficit treatments.

Drought stress resulted in visibly wilted plants exhibiting reduced growth, leaf chlorosis, and progressive necrosis compared to well-watered control plants. Notably, seed priming with 10 and 20 mM sodium silicate visibly alleviated drought-induced phenotypic injury, regardless of the applied Si concentration. Indeed, silicon-primed plants displayed visibly improved growth, well-developed leaves, and markedly improved overall vigor relative to drought-stressed, unprimed plants. Furthermore, well-watered plants primed with 10 and 20 mM Si exhibited enhanced growth compared to unprimed control plants, with notable improvements in shoot length and overall size (Figure 1).

### 2.2. Agronomic Growth Assessment

**Figure 2:**
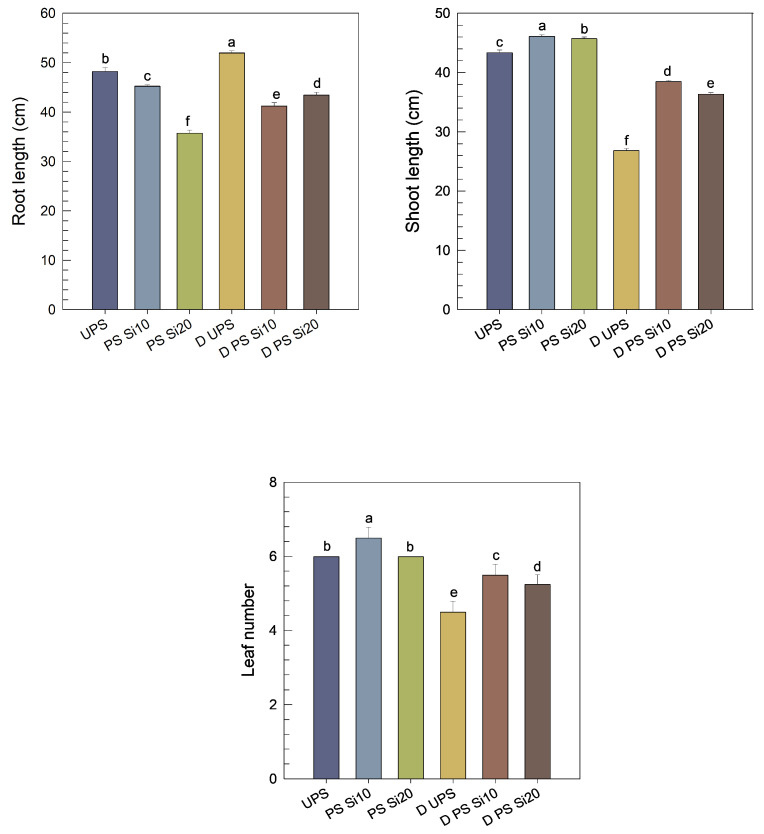
Effects of silicon seed priming on root length, shoot length, and leaf number in maize plants subjected to water deficit. UPS: unprimed seeds; PS Si 10 and PS Si 20: silicon seed priming at 10 and 20 mM; D UPS: unprimed seeds under water deficit; D PS Si 10 and D PS Si 20: silicon seed priming at 10 and 20 mM under water-deficit conditions. Values are presented as means ± standard error (SE) of five replicates. Significant differences among treatments were determined using Duncan’s multiple range tests at *p* < 0.05 for each parameter. Bars denoted by the same letter do not differ significantly.

Under drought stress, unprimed plants exhibited a significant increase in root length, together with marked reductions in shoot length and leaf number relative to well-watered control plants (Figure 2), consistent with a drought-induced increase in main root length and shoot-growth restriction. Silicon seed priming modulated this response by reducing excessive root elongation while improving shoot length and leaf number, suggesting partial alleviation of drought constraints rather than a simple stimulation of root elongation. When comparing the two Si priming concentrations, a priming-solution concentration-dependent response was observed, whereby the 10 mM Si treatment yielded the shortest root length but, conversely, the greatest shoot length and leaf number among drought-stressed plants. Under well-watered conditions, priming with 10 and 20 mM Si resulted in a significant decrease in root length and a significant increase in shoot length and leaf number relative to unprimed control plants (Figure 2).

### 2.3. Fresh Weight-Based Biomass Traits

**Figure 3:**
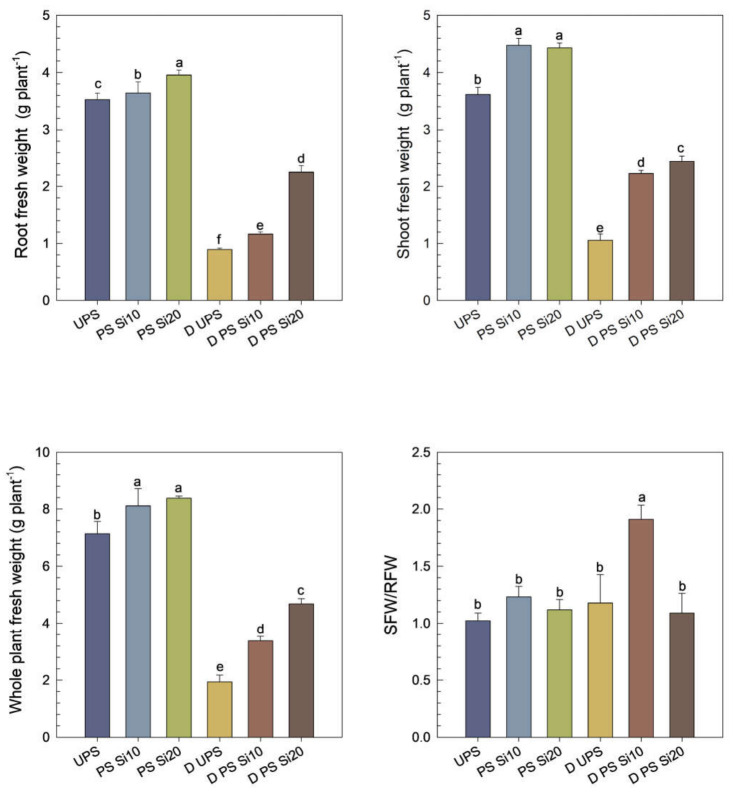
Effects of silicon seed priming on root fresh weight, shoot fresh weight, whole-plant fresh weight, and shoot/root fresh weight ratio (SFW/RFW) of maize plants subjected to water deficit. UPS: unprimed seeds; PS Si 10 and PS Si 20: silicon seed priming at 10 and 20 mM; D UPS: unprimed seeds under water deficit; D PS Si 10 and D PS Si 20: silicon seed priming at 10 and 20 mM under water-deficit conditions. Values are presented as means ± standard error (SE) of five replicates. Significant differences among treatments were determined using Duncan’s multiple range tests at *p* < 0.05 for each parameter. Bars denoted by the same letter do not differ significantly.

As shown in Figure 3, root and shoot fresh weights decreased significantly under drought stress by 75% and 71%, respectively, reflecting a marked decline in fresh biomass production. Silicon seed priming, however, resulted in improved fresh biomass production. Specifically, priming with 10 mM Si yielded a 30% increase in root fresh weight and a 110% increase in shoot fresh weight relative to drought-stressed, unprimed plants. Priming with 20 mM Si produced a more pronounced effect, with root fresh weight increasing by 151% and shoot fresh weight by 131% compared with drought-stressed, unprimed plants. Under well-watered conditions, silicon seed priming also resulted in enhanced root and shoot fresh biomass relative to unprimed control plants. The SFW/RFW ratio also varied among treatments, indicating changes in shoot-to-root fresh biomass allocation. Under water-deficit conditions, the highest SFW/RFW ratio was observed in plants primed with 10 mM Si, whereas plants primed with 20 mM Si showed a ratio closer to that of drought-stressed, unprimed plants, suggesting a more balanced recovery of root and shoot fresh biomass.

### 2.4. Photosynthetic Pigment Contents

**Figure 4:**
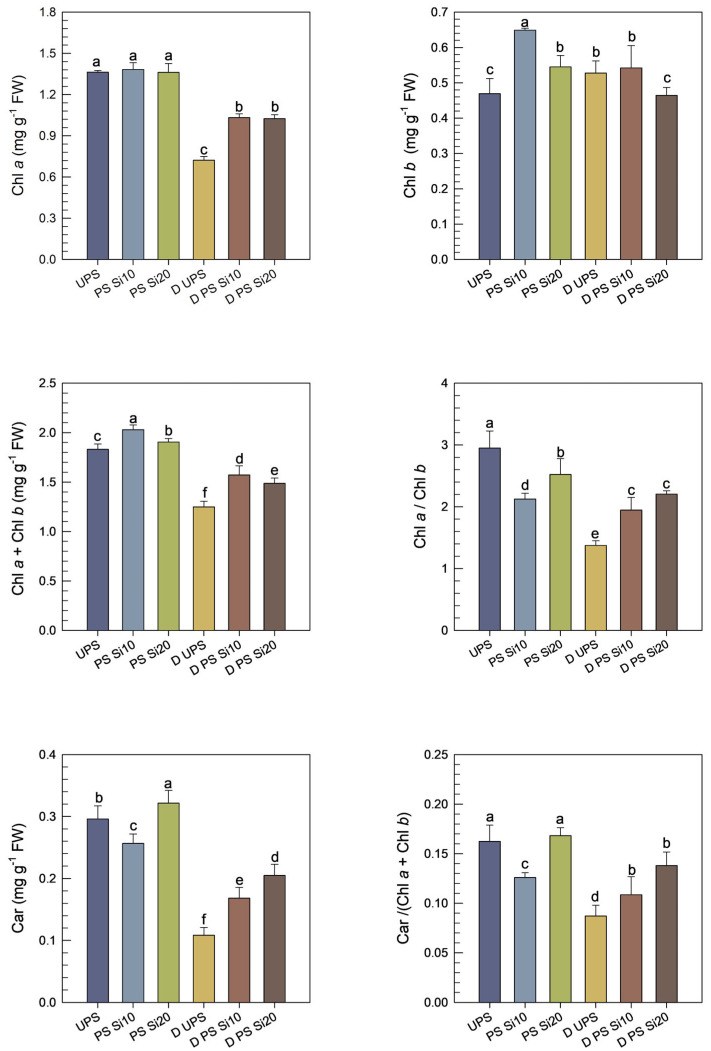
Photosynthetic pigment contents under water-deficit conditions with or without silicon seed priming. Chl *a*: chlorophyll *a* content; Chl *b*: chlorophyll *b* content; Car: carotenoid content; Chl *a* + Chl *b*: total chlorophyll content; Car/(Chl *a* + Chl *b*): ratio of carotenoids to total chlorophyll; Chl *a*/Chl *b*: chlorophyll *a* to chlorophyll *b* ratio. UPS: unprimed seeds; PS Si 10 and PS Si 20: silicon seed priming at 10 and 20 mM; D UPS: unprimed seeds under water deficit; D PS Si 10 and D PS Si 20: silicon seed priming at 10 and 20 mM under water-deficit conditions. Values are presented as means ± standard error (SE) of five replicates. Significant differences among treatments were determined using Duncan’s multiple range tests at *p* < 0.05 for each parameter. Bars denoted by the same letter do not differ significantly.

Under drought stress, plants exhibited a significant reduction in Chl *a*, total chlorophyll (Chl *a* + Chl *b*), and carotenoid (Car) contents, as well as in the Chl *a*/Chl *b* and Car/(Chl *a* + Chl *b*) ratios, by 47%, 32%, 59%, 53%, and 46%, respectively, relative to well-watered control plants (Figure 4).

Silicon seed priming increased several photosynthetic pigment-related parameters under drought stress. Priming with 10 and 20 mM Si increased Chl *a* content by 43% and 42%, total chlorophyll (Chl *a* + Chl *b*) content by 26% and 19%, and Car content by 55% and 88%, respectively, relative to drought-stressed, unprimed plants, indicating a parameter-dependent effect of Si on photosynthetic pigment restoration. Furthermore, the Chl *a*/Chl *b* and Car/(Chl *a* + Chl *b*) ratios were also significantly increased relative to drought-stressed plants, with no significant difference observed between the two applied Si concentrations.

### 2.5. Leaf Relative Water Content

**Figure 5:**
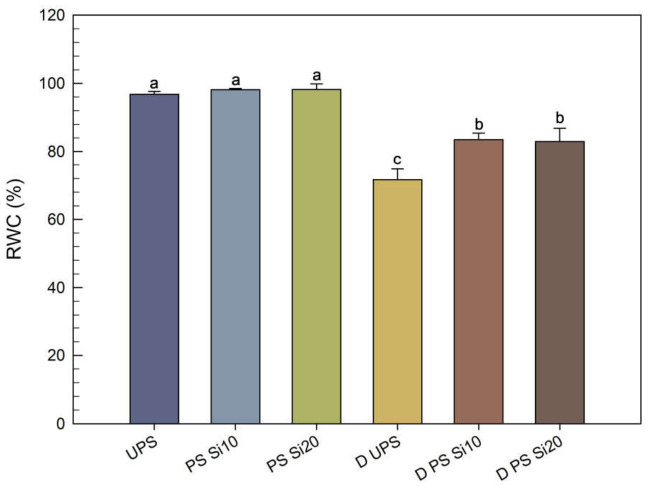
Effects of silicon seed priming on leaf relative water content (RWC) of maize plants subjected to water deficit. UPS: unprimed seeds; PS Si 10 and PS Si 20: silicon seed priming at 10 and 20 mM; D UPS: unprimed seeds under water deficit; D PS Si 10 and D PS Si 20: silicon seed priming at 10 and 20 mM under water-deficit conditions. Values are presented as means ± standard error (SE) of five replicates. Significant differences among treatments were determined using Duncan’s multiple range tests at *p* < 0.05 for each parameter. Bars denoted by the same letter do not differ significantly.

Leaf relative water content (RWC) decreased significantly by 25% under drought stress relative to well-watered control plants, reflecting marked tissue dehydration (Figure 5). This decrease in RWC, together with visible wilting, reduced growth, and pigment loss, supported the conclusion that the irrigation-withholding treatment induced physiological water deficit. Silicon seed priming with both 10 and 20 mM Si partially improved leaf water status, yielding a 17% increase in RWC relative to those of drought-stressed, unprimed plants. No significant difference in RWC was observed between the two applied Si concentrations.

### 2.6. PSII and PSI Photochemical Activity Evaluation

**Figure 6:**
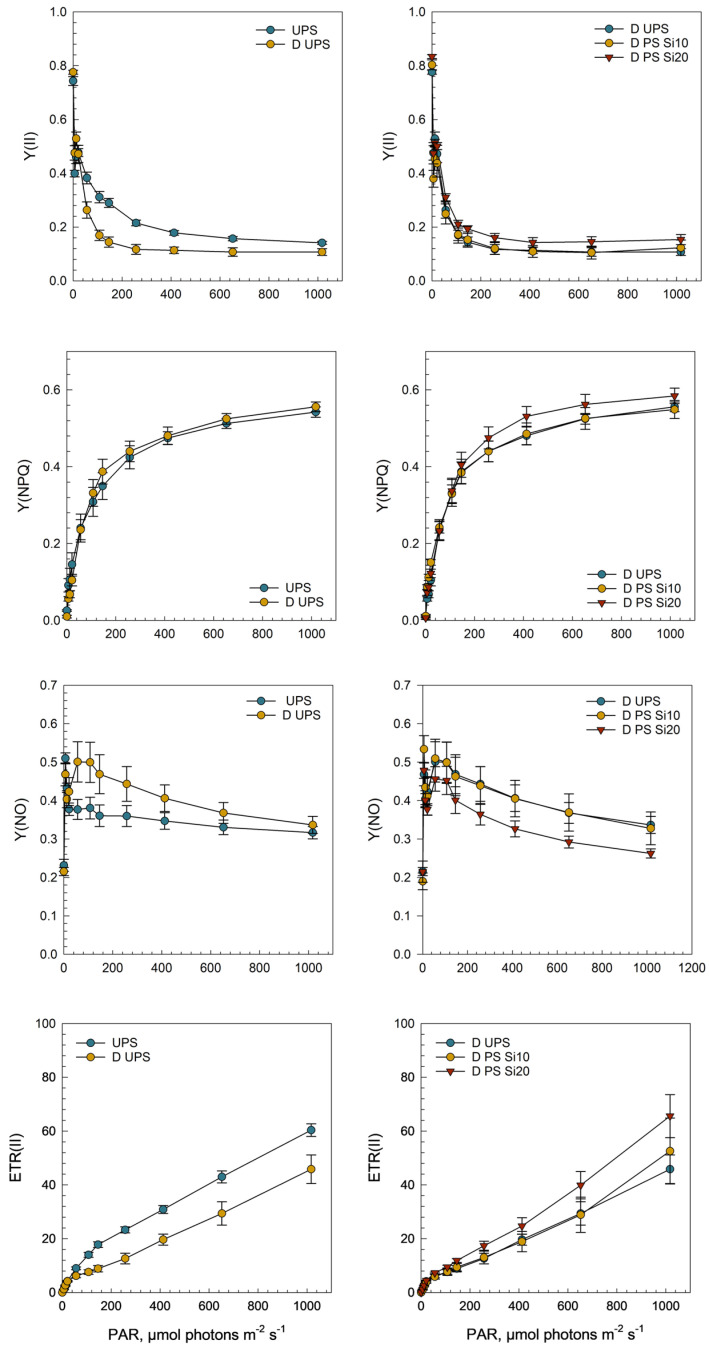
Quantum yields of PSII under water-deficit conditions with or without silicon seed priming. Y(II): quantum yield of photochemical energy conversion in PSII; Y(NPQ): quantum yield of regulated non-photochemical energy dissipation in PSII; Y(NO): quantum yield of non-regulated non-photochemical energy dissipation in PSII; PAR: photosynthetically active radiation. UPS: unprimed seeds; PS Si 10 and PS Si 20: silicon seed priming at 10 and 20 mM; D UPS: unprimed seeds under water deficit; D PS Si 10 and D PS Si 20: silicon seed priming at 10 and 20 mM under water-deficit conditions. Values are presented as means ± standard error (SE) of five replicates.

**Figure 7:**
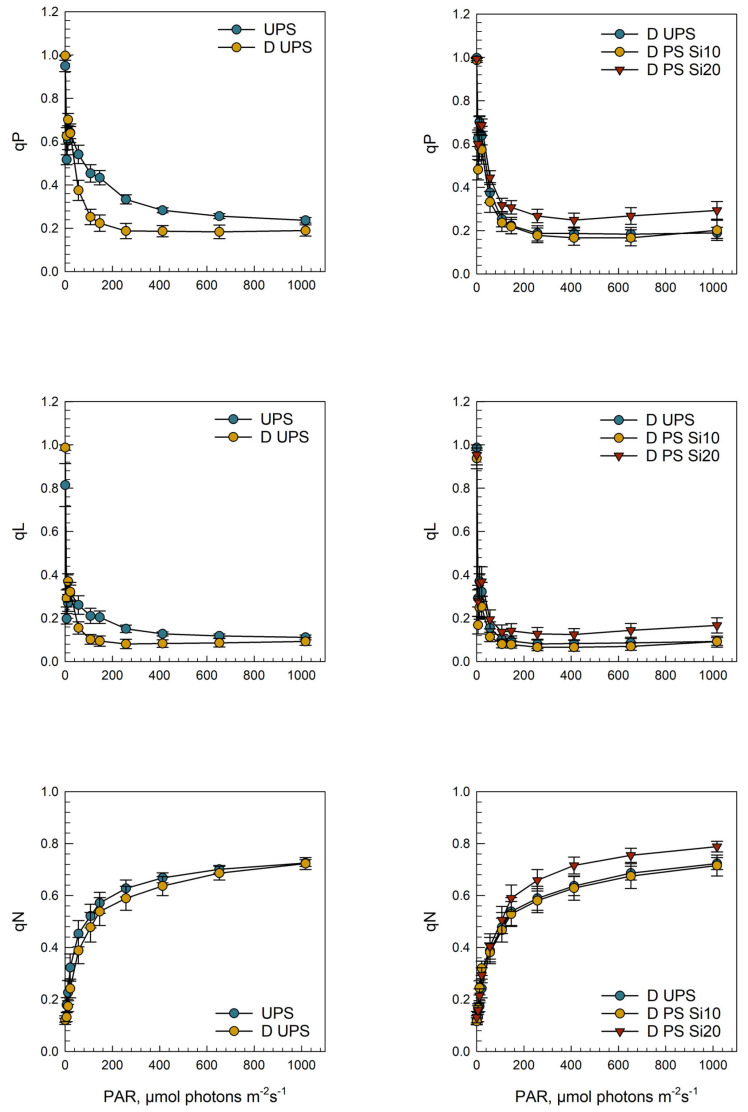
PSII photochemical coefficients under water-deficit conditions with or without silicon seed priming. qP: photochemical quenching coefficient; qL: fraction of open PSII reaction centers; qN: non-photochemical quenching coefficient; PAR: photosynthetically active radiation. UPS: unprimed seeds; PS Si 10 and PS Si 20: silicon seed priming at 10 and 20 mM; D UPS: unprimed seeds under water deficit; D PS Si 10 and D PS Si 20: silicon seed priming at 10 and 20 mM under water-deficit conditions. Values are presented as means ± standard error (SE) of five replicates.

**Figure 8:**
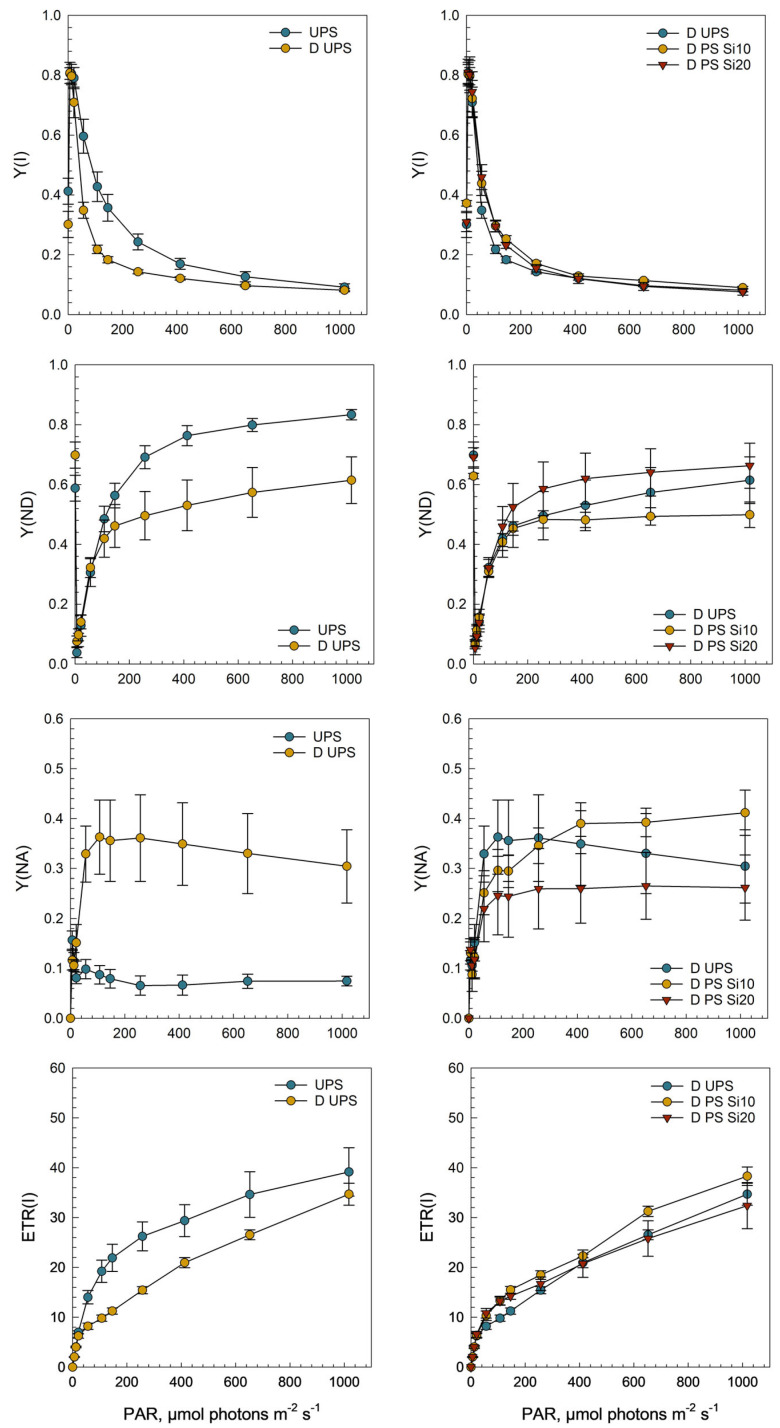
Quantum yields and oxidation state of PSI under water-deficit conditions with or without silicon seed priming. Y(I): quantum yield of photochemical energy conversion in PSI; Y(ND): quantum yield of non-photochemical energy dissipation in reaction centers limited by the donor side; Y(NA): quantum yield of non-photochemical energy dissipation in reaction centers limited by the acceptor side; ETR(I): electron transport rate in PSI; PAR: photosynthetically active radiation. UPS: unprimed seeds; PS Si 10 and PS Si 20: silicon seed priming at 10 and 20 mM; D UPS: unprimed seeds under water deficit; D PS Si 10 and D PS Si 20: silicon seed priming at 10 and 20 mM under water-deficit conditions. Values are presented as means ± standard error (SE) of five replicates.

The photochemical activities of PSII and PSI were also evaluated in the present study (Figure 6, Figure 7 and Figure 8). Regarding PSII, drought stress markedly reduced the effective quantum yield of PSII photochemistry (Y(II)) and the electron transport rate (ETR(II)) across increasing PAR intensities relative to well-watered control plants (Figure 6), reflecting an overall decline in PSII photochemical activity. Concurrently, this decrease was accompanied by a reduction in the yield of regulated non-photochemical energy dissipation (Y(NPQ)) and, conversely, an increase in the yield of non-regulated non-photochemical energy dissipation (Y(NO)), indicating a loss of regulatory control over energy dissipation in PSII under drought conditions.

Silicon seed priming exerted a restorative effect, most prominently at 20 mM Si, as evidenced by a marked increase in Y(II) and ETR(II) relative to drought-stressed, unprimed plants (Figure 6). This corrective effect was further associated with a pronounced increase in Y(NPQ) and a concurrent reduction in Y(NO) at 20 mM Si. Priming with 10 mM Si did not produce a clear improvement in these parameters relative to drought-stressed plants.

An inhibitory effect of drought stress on PSII was further evidenced by marked declines in the photochemical quenching coefficient (qP), the fraction of open PSII reaction centers (qL), and the non-photochemical quenching coefficient (qN) (Figure 7). Priming with 20 mM Si clearly improved all three parameters, yielding notable increases in qP, qL, and qN relative to drought-stressed plants, whereas priming with 10 mM Si produced no clear improvement.

With respect to PSI photochemical activity, drought stress induced a marked decrease in the effective quantum yield of PSI photochemistry (Y(I)) and the electron transport rate (ETR(I)) relative to well-watered control plants (Figure 8). Furthermore, drought stress resulted in a marked increase in the yield of non-photochemical energy dissipation limited by the acceptor side (Y(NA)) and a concurrent decrease in the yield of non-photochemical energy dissipation limited by the donor side (Y(ND)) (Figure 8). Silicon seed priming with both 10 and 20 mM Si improved Y(I) and ETR(I) relative to drought-stressed plants, with a more pronounced positive effect observed at 10 mM Si. These results indicate a photosystem-specific response to silicon seed priming, with 20 mM Si being more effective in restoring PSII-related photochemical and photoprotective parameters, whereas 10 mM Si showed a stronger effect on selected PSI-related parameters.

### 2.7. Two-Way ANOVA Analysis

To better distinguish the effects of water deficit, silicon seed priming, and their interaction, a two-way ANOVA was performed for the main morphological, biomass-related, photosynthetic pigment, and water-status traits (Table 1). Two-way ANOVA revealed that water treatment significantly affected most measured morphological, biomass-related, pigment, and water-status traits, including root length, shoot length, leaf number, biomass-related parameters, chlorophyll *a*, total chlorophyll, chlorophyll *a*/*b* ratio, carotenoid content, carotenoid-to-total chlorophyll ratio, and relative water content, confirming the strong impact of water deficit on maize growth and physiological performance. Silicon seed priming also exerted significant effects on most variables, particularly growth traits, fresh-weight-based biomass, chlorophyll *a*, chlorophyll *b*, total chlorophyll, carotenoids, carotenoid-to-total chlorophyll ratio, and RWC. Importantly, significant water treatment × silicon seed priming interactions were detected for root length, shoot length, shoot fresh weight, root fresh weight, total fresh biomass, shoot/root fresh weight ratio, chlorophyll *a*, chlorophyll *a*/*b* ratio, and carotenoid content. These significant interactions indicate that the effect of silicon seed priming was dependent on water availability for several key morphological, biomass-related, and photosynthetic pigment traits. In contrast, the interaction was not significant for leaf number, chlorophyll *b*, total chlorophyll, carotenoid-to-total chlorophyll ratio, and RWC, suggesting that the silicon effect on these parameters was either additive or less dependent on the water regime. Overall, these results demonstrate that silicon seed priming does not act solely as a general growth-promoting treatment but also induces trait-specific and water-regime-dependent responses that contribute to drought stress mitigation in maize (Table 1).

### 2.8. Correlation Analysis and Principal Component Analysis (PCA)

To further explore the relationships between silicon seed priming and the measured growth, pigment-related, and water-status traits, treatment-wise Pearson correlation analyses were extended to include silicon-primed plants grown under both well-watered and water-deficit conditions. This analysis included PS Si 10 and PS Si 20 under well-watered conditions, as well as D PS Si 10 and D PS Si 20 under water-deficit conditions, thereby allowing a comparative evaluation of trait associations with silicon priming depending on water availability.

As shown in Table 2, silicon-primed treatments exhibited contrasting correlation patterns under well-watered and water-deficit conditions. Under well-watered conditions, both PS Si 10 and PS Si 20 were negatively correlated with root length. In contrast, positive correlations were observed with shoot length, root fresh weight, and whole-plant fresh weight, although the strength and significance of these associations varied between the two priming-solution concentrations. PS Si 10 was also positively associated with Chl *b*, total chlorophyll content, and the SFW/RFW ratio, whereas PS Si 20 showed stronger positive associations with biomass-related traits, particularly shoot fresh weight and whole-plant fresh weight. These results suggest that, in the absence of drought, silicon seed priming was more closely associated with shoot growth, biomass accumulation, and selected pigment-related traits than with root elongation. Under water-deficit conditions, D PS Si 10 and D PS Si 20 showed strong positive correlations with shoot length, root fresh weight, shoot fresh weight, and whole-plant fresh weight, together with strong negative correlations with root length. These patterns indicate that silicon-primed plants under drought were associated with improved biomass accumulation and reduced drought-induced root elongation.

In addition, both D PS Si 10 and D PS Si 20 were positively correlated with Chl *a* and total chlorophyll content, whereas carotenoid-related traits showed stronger positive associations, particularly under D PS Si 20. Leaf relative water content also showed positive associations with both drought-stressed, silicon-primed treatments, although this correlation was statistically significant only for D PS Si 10. Therefore, the drought-related correlation pattern suggests coordinated changes in growth, fresh biomass, pigment preservation, and leaf water status in silicon-primed plants, while the reduction in root length should be interpreted cautiously as a shift away from drought-induced elongation rather than as a simple inhibition of root development.

Notably, the correlation patterns differed between the two silicon priming-solution concentrations. Under well-watered conditions, PS Si 10 showed stronger positive associations with shoot length, Chl *b*, total chlorophyll content, and the SFW/RFW ratio, whereas PS Si 20 was more closely associated with biomass-related traits. Under water-deficit conditions, D PS Si 20 generally showed stronger positive correlations with fresh biomass and pigment-related traits, whereas D PS Si 10 showed stronger associations with the SFW/RFW ratio and RWC. These treatment-specific patterns support a trait-dependent response to silicon seed priming rather than a uniform response across all measured variables.

Overall, these treatment-related associations were more pronounced under water-deficit conditions than under well-watered conditions, suggesting that silicon seed priming was more strongly associated with coordinated improvements in growth, biomass accumulation, pigment preservation, and leaf water status when plants were exposed to drought stress.

Finally, the extended correlation analysis indicates that silicon seed priming was associated with coordinated but treatment-specific changes in growth, biomass allocation, pigment-related traits, and leaf water status. However, these correlations should be interpreted as descriptive associations and not as causal evidence of silicon action.

**Figure 9:**
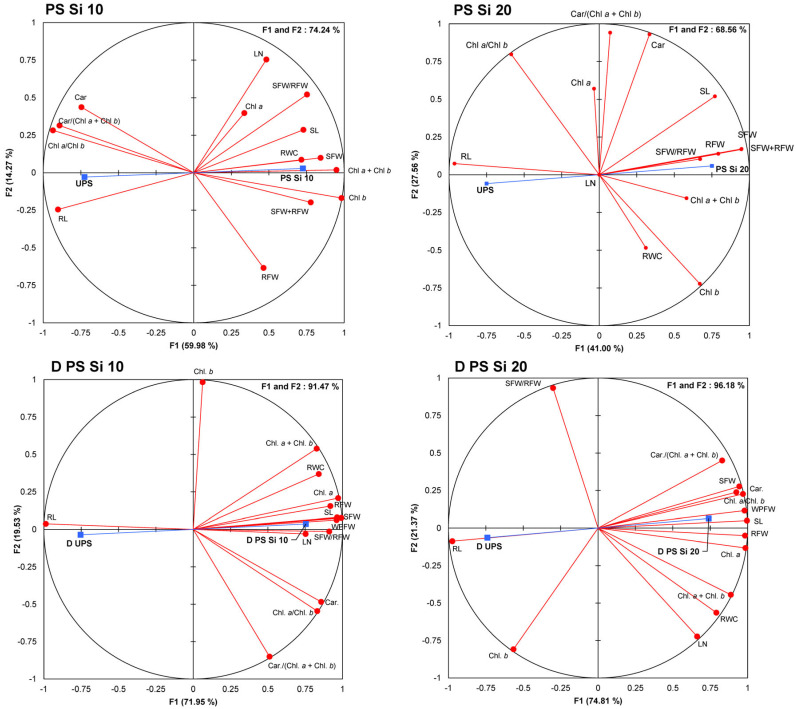
Principal component analysis (PCA) biplots illustrating the relationships among the measured morphological, biomass-related, photosynthetic pigment, and water-status traits in maize plants derived from unprimed seeds and seeds primed with 10 or 20 mM sodium silicate under well-watered and water-deficit conditions. The high cumulative variance explained by the first two principal components should be interpreted with caution because it is influenced by the dimensional structure of the dataset. Therefore, these PCA ordinations are intended as exploratory visualizations of multivariate trait relationships and should be interpreted in conjunction with the two-way ANOVA, univariate analyses, and Pearson correlation analyses. These PCA biplots are presented as descriptive visualizations of treatment-related multivariate patterns and should not be interpreted as independent evidence of causal relationships. Red circles indicate measured variables, whereas blue squares indicate treatment scores. The PCA compares unprimed and silicon-primed plants under well-watered conditions (UPS, PS Si 10, and PS Si 20) and water-deficit conditions (D UPS, D PS Si 10, and D PS Si 20). The F1-F2 planes explained 74.24%, 68.56%, 91.47%, and 96.18% of the total variance for PS Si 10, PS Si 20, D PS Si 10, and D PS Si 20, respectively. Abbreviations: UPS, unprimed seeds under well-watered conditions; PS Si 10 and PS Si 20, silicon-primed seeds with 10 and 20 mM sodium silicate under well-watered conditions; D UPS, unprimed seeds under water-deficit conditions; D PS Si 10 and D PS Si 20, silicon-primed seeds with 10 and 20 mM sodium silicate under water-deficit conditions; RL, root length; SL, shoot length; LN, leaf number; RFW, root fresh weight; SFW, shoot fresh weight; WPFW, whole-plant fresh weight; SFW/RFW, shoot-to-root fresh weight ratio; Chl *a*, chlorophyll *a* content; Chl *b*, chlorophyll *b* content; Chl *a* + Chl *b*, total chlorophyll content; Chl *a*/Chl *b*, chlorophyll *a* to chlorophyll *b* ratio; Car, carotenoid content; Car/(Chl *a* + Chl *b*), carotenoid-to-total chlorophyll ratio; RWC, relative water content.

Following the correlation analysis, PCA was used as a descriptive multivariate approach to visualize the relationships among silicon-primed treatments and the measured growth, biomass-related, pigment-related, and water-status traits (Figure 9). Under well-watered conditions, the F1-F2 plane explained 74.24% and 68.56% of the total variance for PS Si 10 and PS Si 20, respectively. In both biplots, the silicon-primed treatments were separated from the unprimed control, mainly along the F1 axis. For PS Si 10, the positive side of F1 was associated with shoot length, leaf number, shoot fresh weight, the SFW/RFW ratio, Chl *a*, Chl *b*, and total chlorophyll content, whereas root length and carotenoid-related traits were positioned on the negative side of F1. For PS Si 20, the positive side of F1 was mainly associated with biomass-related traits, including root fresh weight, shoot fresh weight, whole-plant fresh weight, and the SFW/RFW ratio, while root length remained positioned opposite to the silicon-primed treatment. These patterns indicate that, under well-watered conditions, silicon seed priming was associated mainly with shoot growth, biomass accumulation, and selected pigment-related responses rather than with root elongation.

Under water-deficit conditions, a clear separation between unprimed and silicon-primed plants was observed. The F1-F2 plane explained 91.47% of the total variance for D PS Si 10 and 96.18% for D PS Si 20. In both drought-related biplots, D UPS was positioned on the negative side of F1, on the same side as root length, whereas D PS Si 10 and D PS Si 20 were positioned on the positive side of F1, together with most growth, biomass, pigment-related, and water-status traits. In the D PS Si 10 biplot, shoot length, root fresh weight, shoot fresh weight, whole-plant fresh weight, Chl *a*, total chlorophyll content, carotenoid content, the Chl *a*/Chl *b* ratio, and RWC were oriented toward the silicon-primed treatment. Similarly, in the D PS Si 20 biplot, the silicon-primed treatment was closely aligned with shoot length, leaf number, root and shoot fresh weights, whole-plant fresh weight, Chl *a*, total chlorophyll, carotenoid content, carotenoid-to-total chlorophyll ratio, and RWC. Conversely, root length was consistently positioned opposite to the silicon-primed treatments, supporting the view that drought-induced root elongation was reduced in primed plants, while biomass accumulation and physiological status were improved.

Overall, the PCA patterns were consistent with the Pearson correlation analysis, showing that silicon seed priming was associated with coordinated changes in growth, fresh biomass, pigment preservation, and leaf water status. These multivariate associations were more evident under water-deficit conditions than under well-watered conditions, particularly for D PS Si 10 and D PS Si 20. Given the limited number of observations included in each PCA, the high cumulative variance explained by the first two principal components should be interpreted cautiously. Therefore, these ordinations are intended as exploratory visualizations of multivariate trait relationships and should be interpreted in conjunction with the two-way ANOVA, univariate analyses, and Pearson correlation analyses.

**Figure 10:**
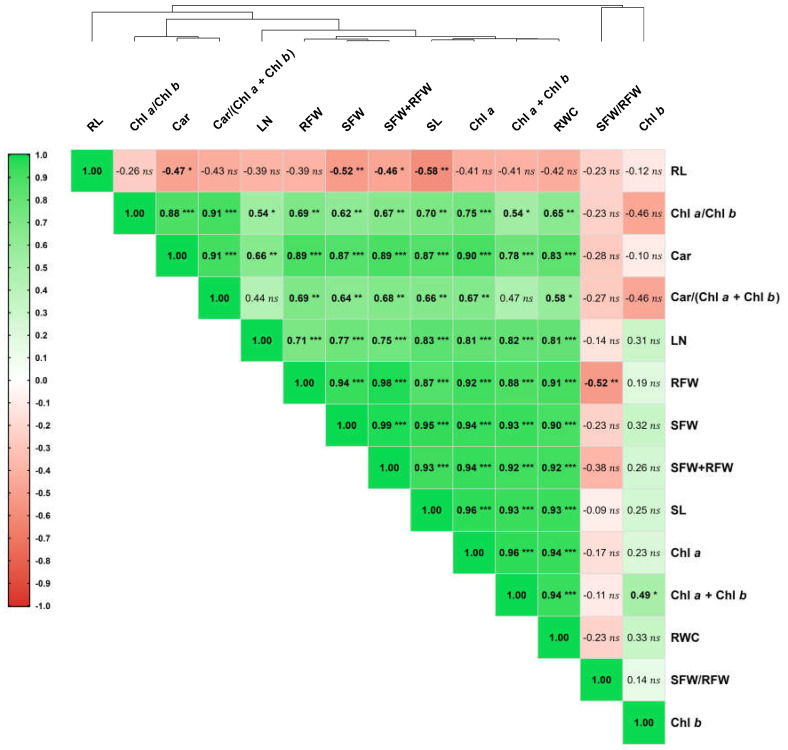
Pearson correlation matrix with hierarchical clustering among morphological, biomass-related, photosynthetic pigment, and water-status traits in maize plants subjected to well-watered and water-deficit conditions, with or without silicon seed priming. Correlation coefficients were calculated using the complete dataset across all six treatments. Variables were reordered according to the similarity of their correlation profiles, as shown by the dendrogram above the matrix. Positive and negative correlations are represented by green and red colors, respectively, with color intensity proportional to correlation strength. Cell values indicate Pearson correlation coefficients (*r*). Except for diagonal self-correlations, bold values indicate statistically significant correlations. Significance levels are indicated as follows: * *p* < 0.05; ** *p* < 0.01; *** *p* < 0.001; *ns*, not significant (*p* ≥ 0.05). Abbreviations: RL: root length; SL: shoot length; LN: leaf number; SFW: shoot fresh weight; RFW: root fresh weight; SFW + RFW: total fresh biomass; SFW/RFW: shoot/root fresh weight ratio; Chl *a*: chlorophyll *a* content; Chl *b*: chlorophyll *b* content; Chl *a* + Chl *b*: total chlorophyll content; Chl *a*/Chl *b*: chlorophyll *a*/chlorophyll *b* ratio; Car: carotenoid content; Car/(Chl *a* + Chl *b*): carotenoid-to-total chlorophyll ratio; RWC: relative water content.

To provide a broader overview of trait coordination across the six treatments and to complement the drought-focused correlation analysis and PCA, a global Pearson correlation analysis was performed using the complete experimental dataset. The analysis examined relationships among morphological, biomass-related, photosynthetic pigment, and water-status traits (Figure 10). In the clustered correlation matrix, variables were reordered according to the similarity of their correlation profiles, as illustrated by the dendrogram above the matrix. This clustering was used only as a visual aid to facilitate interpretation of trait organization and did not modify the underlying Pearson correlation coefficients.

Overall, the correlation matrix revealed strong and biologically coherent associations between shoot growth, fresh-weight-based biomass accumulation, pigment preservation, and plant water status. Shoot length was positively correlated with leaf number, shoot fresh weight, root fresh weight, total fresh biomass, chlorophyll a, total chlorophyll, carotenoid content, and relative water content. These correlations indicate that improved aerial growth was closely associated with higher biomass production, better maintenance of photosynthetic pigments, and improved leaf water status across the complete dataset.

Biomass-related traits, particularly shoot fresh weight, root fresh weight, and total fresh biomass, were also positively associated with chlorophyll a, total chlorophyll, carotenoids, and relative water content. The dendrogram further supported this pattern by grouping several growth, biomass, pigment, and water-status traits within the same correlation module, suggesting a coordinated response among these morphological and physiological parameters.

In contrast, root length showed negative correlations with several shoot- and pigment-related traits, including shoot length, shoot fresh weight, total fresh biomass, and carotenoid content. Its separation from the main growth-biomass-pigment cluster suggests that root elongation followed a distinct response pattern and may reflect an adaptive foraging or drought-avoidance strategy rather than a direct indicator of overall plant vigor. The shoot-to-root fresh weight ratio and chlorophyll *b* content were also less integrated within the main cluster, indicating partially distinct trait dynamics.

The overall correlation pattern indicates that improved drought performance in silicon-primed plants was associated with coordinated changes in shoot growth, biomass maintenance, photosynthetic pigment preservation, and water-status regulation. However, these relationships should be interpreted as associative rather than causal, as correlation analysis does not establish direct mechanistic links among traits.

## 3. Discussion

Drought stress markedly disrupted maize morphology, as evidenced by plant wilting, reduced overall growth, and severe leaf chlorosis progressing to necrosis. Such morphological alterations are characteristic indicators of water deficit and reflect the impairment of cell expansion, photosynthetic efficiency, and nutrient homeostasis under conditions of limited water availability [25,26].

Notably, silicon seed priming at concentrations of 10 and 20 mM substantially mitigated the morphological damage induced by drought stress. Plants subjected to silicon seed priming displayed enhanced growth, more fully developed leaves, and improved overall vigor relative to unprimed, drought-stressed plants, regardless of the applied Si concentration. These findings indicate that silicon seed priming reduced visible drought injury and helped maintain growth traits, fresh biomass, leaf water status, photosynthetic pigment content, and photosystem photochemical activity under water-deficit conditions. Comparable improvements in shoot growth, biomass accumulation, leaf development, and water status following silicon application have been documented in maize, wheat, fenugreek and other crop species under drought stress [10,27,28].

The beneficial effects of silicon on plant morphology under drought conditions have been attributed to a range of physiological and structural mechanisms [23]. Silicon contributes to cell wall reinforcement and overall structural stability, enabling more effective adaptive responses to environmental stresses [13,29]. Recent evidence also highlights plant cell walls as important targets for stomatal regulation, photosynthesis optimization, and improved water-use efficiency [30]. Furthermore, silicon has been reported to enhance hydraulic conductance, allowing plants to maintain water uptake under water-deficit conditions, in contrast to silicon-deficient plants [31]. Collectively, these mechanisms contribute to sustained growth performance and delayed senescence in silicon-primed plants exposed to drought stress.

Consistent with previous reports, silicon seed priming also promoted growth under well-watered conditions, as evidenced by increased shoot length and overall biomass relative to unprimed control plants. This observation should not be interpreted as a novel discovery but rather as a confirmation that silicon can exert growth-promoting effects even in the absence of stress, possibly through improved cell-wall stability, water relations, and nutrient-use efficiency [11,32]. Under well-watered conditions, the response to the two Si priming concentrations was trait-specific, with significant differences for root length and shoot length, whereas other morphological traits showed comparable or parameter-dependent responses. Therefore, low priming-solution concentrations may be sufficient for some, but not all, morphological benefits in maize. The novelty of the present study lies mainly in the integrated evaluation of growth, fresh biomass, leaf water status, pigment preservation, and detailed photosystem photochemical activity in response to silicon seed priming under water-deficit conditions.

Drought stress markedly reduced fresh-weight-based biomass, as evidenced by substantial decreases in root and shoot fresh weight by 75% and 71%, respectively. Because fresh biomass is strongly influenced by tissue water content as well as structural biomass, these changes should not be interpreted solely as dry-matter allocation. Under drought, inhibition of shoot growth represents a major adaptive response because it reduces leaf expansion, transpiring surface, and whole-plant water consumption, while changes in root traits may contribute to maintaining water acquisition. These findings are consistent with previous reports demonstrating that drought stress significantly impairs maize growth, leading to reductions in shoot length as well as shoot and root dry weights [33].

Silicon seed priming significantly mitigated drought-induced fresh biomass loss, highlighting its protective role in maintaining plant growth under water-deficit conditions. Priming with 10 mM Si resulted in a moderate increase in root fresh weight (+30%) but induced a pronounced enhancement of shoot biomass (+110%) relative to drought-stressed, unprimed plants. This substantial stimulation of shoot growth suggests that silicon primarily alleviated drought-related constraints on leaf water status and photosystem photochemical performance rather than directly promoting root development at this concentration. Similar responses have been reported previously, where silicon application enhanced plant growth, biomass accumulation, and photosynthetic pigment content under drought stress conditions [34]. Increasing the priming-solution concentration to 20 mM resulted in a stronger stimulation of root fresh biomass (+151%), indicating a priming-solution concentration-dependent response that may have favored increased root fresh weight rather than primary root elongation. Accordingly, the increase in root fresh weight should not be interpreted as definitive evidence of dry matter accumulation, since fresh weight may be influenced by both structural biomass and tissue water content. In addition, silicon accumulation in plant tissues was not quantified; therefore, the observed differences between the 10 and 20 mM priming treatments should be interpreted as responses to the sodium silicate concentration used during seed priming, rather than as evidence of a confirmed internal Si dose–response relationship. Thus, the shorter root length observed in silicon-primed plants should not be interpreted as a lack of drought adaptation. Rather, it may indicate that silicon seed priming partially reduced drought intensity at the plant level, allowing better maintenance of shoot growth and fresh biomass while limiting the need for excessive primary root elongation. Consistent with these findings, previous studies have reported a significant enhancement of root growth following silicon application in wheat and sorghum under drought stress conditions [35,36]. This response may be related to previously reported roles of silicon in improving plant water uptake under water-limited and salt-stress conditions, including effects on osmotic adjustment [37] and aquaporin expression [38]. However, osmotic adjustment and aquaporin expression were not directly assessed in the present study and should therefore be considered putative mechanisms. Furthermore, the positive effects of silicon on both shoot and root growth in maize may be related to its capacity to enhance photosynthetic activity, thereby promoting overall plant growth, and to reduce stomatal transpiration, leading to improved water-use efficiency under drought stress conditions [30,31,39].

The SFW/RFW ratio further indicated that silicon seed priming modified shoot-to-root fresh biomass allocation under water-deficit conditions. The higher SFW/RFW ratio observed with 10 mM Si suggests a preferential recovery of shoot fresh biomass relative to root fresh biomass, whereas 20 mM Si promoted a more balanced increase in both root and shoot fresh weights.

Previous studies have reported that silicon application can improve root functioning, water management, and oxidative-stress mitigation under drought conditions [40]. However, antioxidant enzyme activities, including catalase activity, were not measured in the present study; therefore, the root fresh-weight responses observed here should not be attributed to catalase activation. Instead, they should be interpreted cautiously as fresh-weight-based responses that may reflect changes in tissue hydration, root morphology, and overall plant water status.

Because only main/longest root length and fresh weight were measured, the present experiment does not allow a mechanistic discrimination among root thickening, increased lateral branching, altered root tissue hydration, or changes in total root-system size. Future root phenotyping using root scanning/image analysis should include root diameter, lateral-root number and branching, total root length, root surface area, and root volume to resolve the shorter-but-heavier root phenotype observed in silicon-primed plants under drought.

The marked decrease in photosynthetic pigments observed in drought-stressed plants confirms the well-documented negative impact of water deficit on the photosynthetic apparatus. The significant reductions in Chl *a*, total chlorophyll (Chl *a* + Chl *b*), and carotenoid contents may reflect impaired pigment biosynthesis and/or accelerated pigment degradation, consistent with oxidative pressure commonly reported under water-deficit conditions. These results are consistent with earlier studies demonstrating that drought stress commonly leads to a reduction in chlorophyll content, which is considered a typical indicator of oxidative damage resulting from pigment photo-oxidation and chlorophyll degradation [25]. Variations in chlorophyll levels under water deficit have been reported across numerous plant species, depending on drought severity and duration [41]. Significant declines in Chl *a*, Chl *b*, and total chlorophyll have been documented in maize, sunflower, and olive under reduced water availability [42,43,44]. This loss of photosynthetic pigments is often linked to structural damage to chloroplasts, including membrane disruption and lamellar disorganization [45], ultimately limiting photosynthetic capacity and primary productivity, with pigment degradation likely associated with chloroplast structural and functional alterations. The decreases in Car/(Chl *a* + Chl *b*) and Chl *a*/Chl *b* ratios further suggest alterations in the organization of light-harvesting complexes and a reduced photoprotective capacity. For example, in wheat, drought-induced changes in chlorophyll composition, including variations in the Chl *a*/Chl *b* ratio, have been reported to differ among cultivars with contrasting drought responses, suggesting that the maintenance of photosynthetic pigment balance may contribute to drought tolerance [46].

The improvement in photosynthetic pigment traits observed in maize plants derived from silicon-primed seeds suggests that Si contributed to pigment preservation, although the statistical interpretation differed among pigment variables. According to the two-way ANOVA, Chl *a* and carotenoid contents showed significant water treatment × Si seed priming interactions, supporting a water-regime-dependent response. In contrast, total chlorophyll (Chl *a* + Chl *b*) showed significant main effects of water treatment and Si seed priming but a non-significant interaction, indicating that the increase in total chlorophyll is better interpreted as an additive priming-related effect rather than as a strictly drought-specific mitigation response. Consistent with this interpretation, previous studies have reported that silicon can limit pigment degradation and improve Chl *a*, Chl *b*, total chlorophyll, and carotenoid contents in plants exposed to water-deficit or comparable stress conditions, including sorghum [47], haricot bean [48], and sea barley [12].

Relative water content (RWC) is considered a reliable physiological indicator of plant water status, as it is closely associated with cell turgor and stomatal conductance [49]. The significant decrease in RWC observed under water-deficit conditions indicates pronounced dehydration of leaf tissues and reflects a disruption of cellular water balance. Comparable reductions in RWC under drought stress have been reported in several studies [50,51].

In contrast, the significant improvement in RWC observed in plants derived from silicon-primed seeds indicates a protective role of Si in maintaining leaf water status under water-deficit conditions. The 17% increase in RWC relative to drought-stressed plants suggests that Si contributes to reducing water loss and enhancing the capacity of leaves to maintain cellular turgor. Karimi and Zare [52] reported that the RWC of silicon-primed melon plants under water-deficit conditions was comparable to that of well-watered control plants, indicating the effectiveness of silicon in maintaining plant water status during drought stress.

To better understand maize responses to silicon seed priming under water-deficit conditions, photosynthetic status was assessed by evaluating the functional activities of PSI and PSII. This assessment is supported by previous studies showing that chlorophyll fluorescence parameters provide valuable information on PSII photochemistry, linear electron transport, and non-photochemical energy dissipation [53,54]. The significant reductions in the effective quantum yield of PSII photochemistry (Y(II)) and the electron transport rate (ETR(II)) observed under drought stress reflect severe impairment of PSII photochemical efficiency. This inhibition was particularly pronounced with increasing photosynthetically active radiation (PAR) intensity, suggesting an inability of the photosynthetic apparatus to process excess light energy under water-deficit conditions. These results are consistent with those of Yasin et al. [55], who demonstrated that water deficit in maize led to a significant reduction in chlorophyll fluorescence parameters, indicative of damage to photosystem II. Under water stress, stomatal closure is commonly reported to limit CO_2_ uptake, thereby reducing photosynthetic carbon assimilation. This decrease in CO_2_ fixation results in a lowered electron transport rate (ETR), while absorbed light energy exceeds the capacity available for photochemistry, causing accumulation of reactive oxygen species (ROS) and inducing oxidative stress [56,57].

The observed decrease in the yield of regulated non-photochemical energy dissipation (Y(NPQ)) and the concomitant increase in non-regulated energy dissipation (Y(NO)) indicate impaired regulated photoprotection and enhanced non-regulated energy dissipation under drought stress [58]. The Y(NPQ) parameter reflects the controlled activation of thermal energy dissipation, primarily mediated by the trans-thylakoid pH gradient and the xanthophyll cycle, notably zeaxanthin [59]. Kalal et al. [60] reported that, in wheat under water stress, an increase in Y(NO) was accompanied by a concomitant increase in Y(NPQ). An earlier study demonstrated that Arabidopsis responds to short-term stress by increasing NPQ, which dissipates excess absorbed light energy as heat, thereby reducing the efficiency of photosynthetic photochemical reactions [61]. Furthermore, a long-term water deficit leads to an increase in PsbS protein content, consistent with enhanced non-photochemical quenching (NPQ) [62]. Since the efficiency of photochemical energy conversion in PSII is directly linked to linear electron transport [63], the reductions in Y(II) and ETR(II) observed under drought stress were associated with a concomitant increase in Y(NO), reflecting enhanced non-regulated energy dissipation and impaired PSII functionality [60].

Silicon seed priming in maize markedly alleviated drought-induced alterations in PSII activity, particularly at the 20 mM concentration, as suggested by marked increases in Y(II) and ETR(II) relative to drought-stressed plants. The recovery of photochemical performance suggests that silicon seed priming may contribute to maintaining the functional integrity of PSII reaction centers and sustaining photosynthetic electron transport under water-deficit conditions. Furthermore, the increase in Y(NPQ), concomitant with the decrease in Y(NO), observed in plants primed with 20 mM Si suggests a partial restoration of regulated thermal energy dissipation mechanisms. The Y(NPQ) parameter is widely recognized as a key indicator of regulated non-photochemical energy dissipation, reflecting the controlled thermal dissipation of excess excitation energy, which plays a fundamental role in safeguarding the photosynthetic apparatus against photodamage under water-deficit conditions [64]. These findings suggest that silicon enhances photoprotective processes by promoting the controlled dissipation of excess excitation energy as heat, thereby preventing PSII overexcitation and reducing the risk of photodamage. Similar results were reported by Kalal et al. [60] in wheat subjected to water deficit and primed with SiO_2_ nanoparticles, confirming the protective effect of silicon on photosystems under drought stress.

Water deficit significantly impaired the qP, qL, and qN parameters. The decline in qP observed under water-deficit conditions may reflect reduced photochemical sink capacity and a lower proportion of open PSII reaction centers, processes that are commonly associated with drought-induced limitations in CO_2_ assimilation. The detrimental effects on these photochemical parameters were markedly alleviated by silicon seed priming at 20 mM. Previous studies have demonstrated that interactions between thylakoid membranes and specific protective compounds, which mitigate the impacts of environmental stressors, can induce structural modifications that increase ETR and qP, thereby improving the functional efficiency of the photosynthetic apparatus [65,66]. In agreement, Falouti et al. [67] reported that, under salinity stress, qL, qP, and qN remained largely unchanged, indicating that the photosynthetic machinery maintained a stable fraction of open PSII reaction centers, photochemical quenching, and non-photochemical quenching, respectively.

Under drought conditions, PSI photochemical performance was substantially impaired, as indicated by marked reductions in both the effective quantum yield (Y(I)) and the electron transport rate (ETR(I)) relative to well-watered control plants. This impairment was associated with a pronounced increase in acceptor-side-limited non-photochemical energy dissipation (Y(NA)) and a concomitant decrease in donor-side-limited dissipation (Y(ND)), reflecting a disruption in the balance of energy allocation within PSI.

Drought stress is known to disturb photosynthetic electron transport and photosystem functionality through multiple physiological and biochemical constraints [68]. In the present study, this disturbance was reflected by an increase in Y(NA) and a decrease in Y(ND), indicating enhanced acceptor-side limitation and reduced donor-side limitation of PSI. Such PSI imbalance may be associated with drought-induced impairment of PSII activity, since PSII photoinhibition and its repair cycle play an important role in protecting PSI from irreversible damage [69]. Furthermore, Y(ND) serves as an indicator of donor-side limitations in PSI, linked to non-photochemical energy dissipation at the PSI level [70]. Kalal et al. [60] demonstrated that, under drought stress in wheat, the increase in Y(NA) reflects an acceptor-side limitation of PSI, corresponding to a fraction of P700 that cannot be oxidized due to insufficient electron acceptors. The simultaneous decline in Y(I) and Y(II) further suggests that electron transport through both PSI and PSII was severely constrained under water-deficit conditions, consistent with the observed reductions in ETR(I) and ETR(II).

Silicon seed priming with 10 and 20 mM Si effectively mitigated these inhibitory effects, restoring Y(I) and ETR(I) relative to drought-stressed plants, with the most pronounced improvement observed at 10 mM Si. Notably, the 20 mM Si treatment induced a significant increase in Y(ND) alongside a decrease in Y(NA), indicating that the recovery of PSI quantum yield was associated with enhanced donor-side control and reduced acceptor-side limitation. Collectively, these results suggest a potential role of sodium silicate priming in stabilizing PSI function, maintaining electron transport efficiency, and preserving photochemical energy balance under drought stress conditions. The different responses of PSI and PSII to 10 and 20 mM Si should not be interpreted as contradictory. Rather, they suggest that the two photosystems may have different sensitivity thresholds to the sodium silicate priming-solution concentration. The 20 mM treatment appeared more effective in restoring PSII photochemistry and regulated thermal energy dissipation, whereas the 10 mM treatment showed a stronger effect on PSI photochemical yield and ETR(I). Because tissue Si accumulation and gas-exchange parameters were not measured, this interpretation remains physiological and should be confirmed in future studies.

Taken together, the present findings support a physiological interpretation based on the measured variables, including leaf water-status maintenance, pigment preservation, regulated energy dissipation, and PSI/PSII photochemical stability. However, antioxidant activity, osmotic adjustment, ROS accumulation, gas-exchange parameters, and Si accumulation were not directly measured; therefore, these mechanisms should be considered plausible explanations requiring further experimental confirmation.

## 4. Materials and Methods

### 4.1. Plant Material and Culture Conditions

The experiment was conducted in a greenhouse using a soil-peat substrate and maize (*Zea mays* L.), a species extensively cultivated in Tunisia. Maize seeds were initially screened to exclude any specimens exhibiting physical damage, biological contamination, or morphological abnormalities. The selected seeds were subsequently surface-sterilized by immersion in a 1% sodium hypochlorite (NaClO) solution for 3 min, followed by three rinses with sterile distilled water.

After brief drying at room temperature on sterile filter paper, the seeds were allocated into three experimental groups: one unprimed control group (UPS) and two silicon-primed groups (PS Si 10 and PS Si 20), soaked for 8 h in sodium silicate (Na_2_SiO_3_; Sigma-Aldrich, St. Louis, MO, USA, extra pure) solutions at concentrations of 10 and 20 mM, respectively. These concentrations refer exclusively to the sodium silicate priming solutions and should not be interpreted as measured internal Si concentrations or tissue Si accumulation. The selected Na_2_SiO_3_ concentrations and the 8 h priming duration were chosen based on previous silicon seed-priming studies in maize and related crops in order to provide sufficient seed hydration and exposure to soluble silicon while avoiding excessive imbibition before sowing. Following priming, seeds were air-dried back to approximately their original moisture content, verified by seed weight/moisture measurement, and subsequently sown in plastic pots (12 cm height × 14 cm diameter) filled with 500 g of soil-peat substrate. After 20 days of growth, each treatment group was subdivided into two subgroups, and water stress was imposed by withholding irrigation for 15 days. Thus, the water deficit was operationally imposed by withholding irrigation for 15 days rather than by maintaining a predefined substrate water content or water potential. Soil/substrate water content was not directly monitored during the experiment; therefore, drought intensity was inferred from the imposed irrigation withdrawal and from plant-level responses, including visible wilting, reduced growth, pigment decline, and reduced leaf relative water content. The experiment was conducted over 35 days after sowing under natural light conditions, with temperatures ranging from 23 to 25 °C, in a greenhouse at the Center of Biotechnology of Borj-Cedria, northeastern Tunisia (36°42′32.9″ N, 10°25′40.9″ E).

At harvest, maize plants were separated into roots, stems, and leaves. Roots were gently separated from the soil-peat substrate, and adhering substrate particles were removed by careful rinsing with distilled water. The roots were then gently blotted with filter paper before measurement. Root length was measured manually as the length of the main/longest root axis, from the root–shoot junction to the distal root tip. Therefore, the reported root length does not represent total root-system length or cumulative lateral root length. Root fresh weight, shoot fresh weight, and total fresh weight were recorded, with shoot fresh weight defined as stem + leaf fresh weight.

### 4.2. Experimental Design and Treatments

Six experimental treatments were established to evaluate the effects of silicon seed priming and water availability on maize: well-watered control plants without silicon priming (UPS); well-watered plants primed with 10 mM sodium silicate (PS Si 10) or 20 mM sodium silicate (PS Si 20); drought-stressed control plants without silicon priming (D UPS); and drought-stressed plants primed with 10 mM sodium silicate (D PS Si 10) or 20 mM sodium silicate (D PS Si 20). Pots were randomly distributed within the greenhouse and periodically repositioned during the experiment to minimize potential positional effects related to light, temperature, or airflow gradients.

### 4.3. Photosynthetic Pigment Analysis

Chlorophylls and total carotenoids were extracted from 100 mg of fresh leaf tissue homogenized in 5 mL of 80% acetone and incubated in the dark at 4 °C for 72 h. The absorbance of the resulting pigment extracts was subsequently measured at 663, 646, and 470 nm using a UV–visible spectrophotometer (Specord 210 Plus, Analytik Jena, Jena, Germany). The concentrations of chlorophyll *a* (Chl *a*), chlorophyll *b* (Chl *b*), and total carotenoids (Car) were then calculated according to the equations described by Lichtenthaler and Wellburn [71].

### 4.4. Relative Water Content (RWC)

Relative water content (RWC) was determined on fully expanded leaves by recording fresh weight (FW) immediately after harvest, floating the leaf disks in distilled water for 24 h to obtain turgid weight (TW), and oven-drying them at 80 °C until constant weight to determine dry weight (DW). RWC was calculated according to Barrs and Weatherley [72] as follows: RWC (%) = ((FW − DW)/(TW − DW)) × 100.

### 4.5. Measurements of Photosystem Activities

The functional activities of photosystems I (PSI) and II (PSII) were quantified using a Dual-PAM-100 system (Heinz Walz, Effeltrich, Germany). For all treatments, measurements were performed on the youngest fully expanded and visually healthy leaf borne on the main stem. When more than one leaf fulfilled this criterion, the uppermost fully expanded leaf was selected. This procedure was used to ensure comparable leaf developmental status among plants and to minimize variation related to leaf age or position. Prior to measurements, leaves were dark-adapted for 30 min. Subsequently, actinic light was applied incrementally from 0 to 1017 μmol photons m^−2^ s^−1^ to evaluate electron transport rates and PSII quantum yields, including the effective quantum yield of PSII photochemistry (Y(II)), regulated non-photochemical energy dissipation (Y(NPQ)), and non-regulated non-photochemical energy dissipation (Y(NO)). PSI quantum yields were assessed using a dual-wavelength 830/875 nm emitter-detector system, yielding measurements of the effective quantum yield of PSI photochemistry (Y(I)), donor-side-limited non-photochemical dissipation (Y(ND)), and acceptor-side-limited non-photochemical dissipation (Y(NA)).

### 4.6. Statistical Analysis

Statistical analyses were conducted using a factorial approach consistent with the experimental design. For each response variable, data were analyzed by a two-way analysis of variance (two-way ANOVA) using R software (v. 4.6.0, 2026, R Foundation for Statistical Computing, Vienna, Austria) to test the main effects of water treatment, silicon seed priming, and their interaction (water treatment × silicon seed priming).

When significant effects were detected, treatment means were compared using Duncan’s multiple range test at *p* < 0.05. Duncan’s post hoc comparisons and the corresponding homogeneous group letters were generated using XLSTAT software (v. 2014; Addinsoft, Paris, France) and were independently verified using R software (v. 4.6.0, 2026). Identical post hoc groupings were obtained with both statistical workflows.

Treatment-wise Pearson correlation analyses were performed using XLSTAT software (v. 2014; Addinsoft, Paris, France) to explore descriptive associations between silicon-primed treatments and measured growth, biomass, photosynthetic pigment, and water-status traits under both well-watered and water-deficit conditions.

Principal component analysis (PCA) was performed on standardized variables using XLSTAT software (v. 2014; Addinsoft, Paris, France) as an exploratory multivariate approach to visualize treatment-related patterns and relationships between treatments and measured variables. The PCA biplots were generated at the treatment level and were therefore used as an exploratory multivariate tool to visualize treatment-related trait associations. The explained variance percentages were interpreted in this descriptive context, together with the correlation analyses and univariate statistical results.

In addition to the treatment-based statistical analyses, a global Pearson correlation analysis was performed using R software to examine the overall relationships among measured traits across the complete dataset. Pearson correlation coefficients and their associated *p*-values were calculated using pairwise complete observations. Correlation significance was indicated as *ns*, non-significant (*p* ≥ 0.05); * *p* < 0.05; ** *p* < 0.01; and *** *p* < 0.001. To facilitate interpretation, variables were reordered by hierarchical clustering based on the distance 1 − |r|, and the resulting dendrogram was displayed above the upper-triangular correlation matrix.

## 5. Conclusions

The present study indicates that silicon seed priming can improve maize performance under water-deficit conditions by partially preserving growth, fresh biomass, leaf water status, photosynthetic pigment content, and photosystem functionality. The two-way ANOVA confirmed that several responses to silicon priming were dependent on water availability, particularly for morphological, biomass-related, and selected pigment traits, whereas other traits showed additive or less water-regime-dependent responses. Overall, the effects of silicon seed priming were trait- and photosystem-dependent rather than uniformly dependent on the sodium silicate concentration used for seed priming, with 20 mM Si being particularly effective for biomass recovery and selected PSII-related parameters, whereas 10 mM Si showed stronger effects for selected growth and PSI-related parameters. However, the present interpretation was restricted to the measured parameters, namely growth, fresh biomass, photosynthetic pigments, leaf relative water content, and PSI/PSII photochemical activity.

Future studies should combine these measurements with direct soil/substrate water-content monitoring, antioxidant enzyme activities, ROS-related markers, osmotic adjustment, osmolyte accumulation, gas-exchange parameters, Si uptake/tissue-allocation analyses, and detailed root-system phenotyping, including root diameter, lateral branching, total root length, root surface area, root volume, and root–shoot relationships, to better clarify the physiological, biochemical, and root-architectural mechanisms underlying Si-mediated drought mitigation.

## Figures and Tables

**Table 1:** Two-way ANOVA results for morphological, biomass-related, photosynthetic pigment, and leaf relative water content variables. The two-way ANOVA model included water treatment, silicon seed priming, and their interaction as fixed effects to determine whether the effect of silicon priming depended on water availability. Significance codes: *ns*, not significant (*p* ≥ 0.05); *, *p* < 0.05; **, *p* < 0.01; ***, *p* < 0.001.

Variable	Water Treatment	Si Seed Priming	Water × Si Interaction
RL	*F* = 30.682; *p* < 0.001 ***	*F* = 186.170; *p* < 0.001 ***	*F* = 58.398; *p* < 0.001 ***
SL	*F* = 2244.5; *p* < 0.001 ***	*F* = 353.906; *p* < 0.001 ***	*F* = 132.594; *p* < 0.001 ***
LN	*F* = 33.800; *p* < 0.001 ***	*F* = 5.400; *p* = 0.0146 *	*F* = 1.400; *p* = 0.2722 *ns*
SFW	*F* = 773.775; *p* < 0.001 ***	*F* = 75.083; *p* < 0.001 ***	*F* = 4.097; *p* = 0.0342 *
RFW	*F* = 666.225; *p* < 0.001 ***	*F* = 38.088; *p* < 0.001 ***	*F* = 10.622; *p* < 0.001 ***
SFW + RFW	*F* = 1184.6; *p* < 0.001 ***	*F* = 77.229; *p* < 0.001 ***	*F* = 11.271; *p* < 0.001 ***
SFW/RFW	*F* = 21.309; *p* < 0.001 ***	*F* = 28.618; *p* < 0.001 ***	*F* = 13.153; *p* < 0.001 ***
Chl *a*	*F* = 203.770; *p* < 0.001 ***	*F* = 11.536; *p* = 0.0016 **	*F* = 10.210; *p* = 0.0026 **
Chl *b*	*F* = 1.958; *p* = 0.1870 *ns*	*F* = 4.191; *p* = 0.0417 *	*F* = 2.785; *p* = 0.1015 *ns*
Chl *a* + Chl *b*	*F* = 111.746; *p* < 0.001 ***	*F* = 10.977; *p* = 0.0019 **	*F* = 1.170; *p* = 0.3434 *ns*
Chl *a*/Chl *b*	*F* = 22.309; *p* < 0.001 ***	*F* = 1.707; *p* = 0.2227 *ns*	*F* = 9.195; *p* = 0.0038 **
Car	*F* = 89.724; *p* < 0.001 ***	*F* = 7.460; *p* = 0.0078 **	*F* = 4.568; *p* = 0.0335 *
Car/(Chl *a* + Chl *b*)	*F* = 16.229; *p* = 0.0017 **	*F* = 4.630; *p* = 0.0323 *	*F* = 2.989; *p* = 0.0884 *ns*
RWC	*F* = 99.479; *p* < 0.001 ***	*F* = 5.519; *p* = 0.0200 *	*F* = 3.433; *p* = 0.0662 *ns*

Abbreviations: RL: root length; SL: shoot length; LN: leaf number; SFW: shoot fresh weight; RFW: root fresh weight; Chl *a*: chlorophyll *a* content; Chl *b*: chlorophyll *b* content; Car: carotenoid content; RWC: relative water content. *F* = *F* statistic with numerator and residual degrees of freedom. *p* = probability value.

**Table 2:** Pearson correlation coefficients between measured traits and silicon seed-priming treatments under well-watered and water-deficit conditions. PS Si 10 and PS Si 20 correspond to plants grown under well-watered conditions after seed priming with 10 and 20 mM sodium silicate, respectively. D PS Si 10 and D PS Si 20 correspond to plants grown under water-deficit conditions after seed priming with 10 and 20 mM sodium silicate, respectively. The 10 and 20 mM values refer to the sodium silicate concentrations used in the priming solutions and should not be interpreted as measured internal silicon concentrations. Negative correlations are shown in red, whereas positive correlations are shown in green; color intensity is proportional to the magnitude of the correlation coefficient. Bold values indicate statistically significant correlations. Asterisks indicate significance levels: * *p* < 0.05; ** *p* < 0.01; *** *p* < 0.001; *ns*, not significant (*p* ≥ 0.05).

−1					
−0.9					
−0.8					
−0.7	Variables	PS Si 10	PS Si 20	D PS Si 10	D PS Si 20
−0.6	RL	−0.84 **	−0.98 ***	−0.99 ***	−0.98 ***
−0.5	SL	0.92 **	0.89 **	1.00 ***	0.99 ***
−0.4	LN	0.58 *ns*	0.00 *ns*	0.71 *	0.63 *ns*
−0.3	RFW	0.90 **	0.91 **	0.94 ***	0.98 ***
−0.2	SFW	0.21 *ns*	0.78 *	0.97 ***	0.97 ***
−0.1	WPFW	0.74 *	0.92 **	0.98 ***	0.99 ***
0	SFW/RFW	0.84 **	0.59 *ns*	0.91 **	−0.23 *ns*
0.1	Chl *a*	0.19 *ns*	−0.01 *ns*	0.97 **	0.97 ***
0.2	Chl *b*	0.90 *	0.58 *ns*	0.10 *ns*	−0.61 *ns*
0.3	Chl *a* + Chl *b*	0.82 *	0.51 *ns*	0.84 *	0.85 *
0.4	Chl *a*/Chl *b*	−0.82 *	−0.50 *ns*	0.80 *ns*	0.98 ***
0.5	Car	−0.62 *ns*	0.41 *ns*	0.82 *	0.92 ***
0.6	Car/(Chl *a* + Chl *b*)	−0.74 *ns*	0.16 *ns*	0.46 *ns*	0.84 *
0.7	RWC	0.62 *ns*	0.37 *ns*	0.86 *	0.76 *ns*
0.8					
0.9					
1					

Abbreviations: RL: root length; SL: shoot length; LN: leaf number; Chl *a*: chlorophyll *a* content; Chl *b*: chlorophyll *b* content; Car: carotenoid content; RFW: root fresh weight; SFW: shoot fresh weight; RWC: relative water content.

## Data Availability

All data generated or analyzed during this study are included in this published article. Additional datasets are available from the corresponding author upon a reasonable request.
